# Neurotoxic Effects of Bisphenol (BPA): Mini-Reviews

**DOI:** 10.3390/toxics13100888

**Published:** 2025-10-17

**Authors:** Luciana Veras de Aquino Figueirôa, Tiago da Silva Teófilo, Jael Soares Batista, Ana Caroline Maia Oliveira Ramos, Gustavo Coringa de Lemos, Salvador Viana Gomes Junior, Guilherme Braga Silva Lima, Jose Leonilson Feitosa, Ana Beatriz da Silva, Larissa Nayara de Souza, Roque Ribeiro da Silva Júnior, Maria Irany Knackfuss, Edson Fonseca Pinto, Ellany Gurgel Cosme do Nascimento, Thales Allyrio Araújo de Medeiros Fernandes, Fausto Pierdoná Guzen

**Affiliations:** 1 Postgraduate Program in Animal Science (PPGCA), Federal Rural University of the Semi-Arid Region (UFERSA), Rua Francisco Mota, 572, Costa e Silva, Mossoró 59625-900, RN, Brazil; lucianaveras_1@yahoo.com.br (L.V.d.A.F.); tiago.teofilo@ufersa.edu.br (T.d.S.T.); jael.batista@ufersa.edu.br (J.S.B.); vetcarolmaia@gmail.com (A.C.M.O.R.); 2 Postgraduate Program in Health Biotechnology (PPGB), Potiguar University (UNP), Av. Senador Salgado Filho, 1610, Lagoa Nova, Natal 59056-000, RN, Brazil; gustavocoringafisio@gmail.com; 3 Faculty of Health Sciences, Multicenter Graduate Program in Physiological Sciences (PPGMCF), State University of Rio Grande do Norte (UERN), Rua Prof. Antônio Campos, BR 110, Costa e Silva, Mossoró 59610-090, RN, Brazil; salvajunior@live.com (S.V.G.J.); lfodontologiaeensino@gmail.com (J.L.F.); thalesallyrio@uern.br (T.A.A.d.M.F.); faustoguzen@uern.br (F.P.G.); 4 Postgraduate Program in Health and Society (PPGSS), State University of Rio Grande do Norte (UERN), Mossoró 59607-360, RN, Brazil; guilhermebragasl302@gmail.com (G.B.S.L.); ana20241002010@alu.uern.br (A.B.d.S.); larissanay15@gmail.com (L.N.d.S.); mariaknackfuss@uern.br (M.I.K.); edsonpinto@uern.br (E.F.P.); ellanygurgel@uern.br (E.G.C.d.N.)

**Keywords:** bisphenol A, neurotoxicity, prefrontal cortex, hippocampus

## Abstract

Introduction: Bisphenol A (BPA) is a synthetic compound widely used in plastics and epoxy resins, and human exposure is virtually unavoidable. Numerous studies indicate that even doses below current regulatory limits may elicit neurotoxic effects, impairing learning, memory, and synaptic plasticity. Methodology: This mini-review. Searches were conducted in PubMed, the Virtual Health Library (VHL/BVS), and ScienceDirect, using MeSH descriptors related to “Bisphenol A,” “Neurotoxicity Syndromes,” “Central Nervous System,” and “Prefrontal Cortex,” combined with Boolean operators. We included studies published between 2007 and 2025, available in English, Portuguese, or Spanish, and focused on the neurotoxic effects of BPA. After screening and application of the eligibility criteria, twelve articles were selected. Results: The analyzed studies show that BPA exposure, even at low concentrations, compromises neuronal survival, dendritic density, and synaptic plasticity. In animal models, cognitive deficits were observed in memory and learning tasks, associated with increased oxidative stress and alterations in molecular pathways such as AMPK, HO-1, and nNOS/Keap1/Nrf2. In cell cultures, BPA induced apoptosis, autophagy dysfunction, cytoskeletal reorganization, and loss of synaptic proteins. The effects were dose-dependent and, in some cases, sex-dependent. Conclusions: BPA exhibits significant neurotoxic potential, affecting both the development and function of the central nervous system. These findings underscore the need to revise current safety limits and reinforce the importance of public policies regulating BPA use, as well as encouraging the search for safer alternatives.

## 1. Introduction

Bisphenol A (BPA) is one of the most ubiquitous synthetic chemical compounds in contemporary society and is widely used as a monomer in the production of polycarbonate plastics and epoxy resins materials found in the inner lining of most food cans and in dental sealants. Human exposure to BPA has become virtually unavoidable, as heat and extreme environmental conditions (such as acidic or basic media) accelerate the hydrolysis of the ester bonds linking its monomers, thereby facilitating the release of the substance into foods and beverages and, consequently, increasing the risk of contamination [1].

In parallel with these exposure mechanisms, international regulatory agencies have established safety limits [2]. Reports indicate that the European Food Safety Authority (EFSA) set the tolerable daily intake (TDI) at approximately 0.2 ng/kg body weight per day, a lifetime exposure parameter representing a substantial reduction from the previously adopted limit of up to 4 µg BPA/kg/day.

Even within such safety limits, experimental evidence from animal models shows that BPA exposure can impair brain development, cognitive functions, and behavior and may even be associated with the emergence of mental disorders such as schizophrenia [3,4]. Although much of the literature focuses on neurotoxic effects resulting from perinatal exposure and on mechanisms modulating its impact on neurodevelopment [5], recent studies suggest that BPA also exerts adverse effects on the adult brain, an area for which toxicogenomic evidence remains scarce [6].

Furthermore, several studies indicate that BPA interferes with both reproductive and non-reproductive behaviors, such as playful interactions and performance on learning tasks in female and male rodents. These findings, unexpected for a xenobiotic estrogen, are consistent with observations of dendritic spine synapse loss [7].

Moreover, investigations show that BPA, even at doses below the daily exposure limit recommended by agencies such as the EPA, can impair the synaptogenic response to 17β-estradiol in the hippocampus of ovariectomized female rats [8]. Inhibition of estrogen-induced dendritic spine synaptogenesis is associated with cognitive deficits, particularly in individuals with physiologically reduced levels of this hormone, as observed in postmenopausal women [9].

Taken together, these findings underscore the need for public policies aimed at promoting health and preventing risks associated with BPA exposure through stricter regulations on the production, commercialization, and use of such compounds. In this context, the present study aimed to conduct a mini-review of the literature on the neurotoxic effects of bisphenol A, with an emphasis on potential clinical conditions associated with exposure to this compound.

## 2. Methodology

This mini-review was conducted in a manner similar to that described by Gomes et al. [10]. Searches were performed in PubMed, the Virtual Health Library (VHL/BVS), and ScienceDirect. We predefined a mechanistic scope: inclusion was restricted to primary experimental studies (in vitro/in vivo) that evaluated bisphenol A (BPA) alone (or BPA ± a single modulator when the BPA effect could be isolated) and reported quantitative cellular/synaptic outcomes in neural cells (human or animal, primary cultures or cell lines) and/or brain structures, with emphasis on the prefrontal cortex (PFC) and hippocampus (e.g., neuritogenesis, dendritic spines, synaptic proteins, ROS/RNS, apoptosis/autophagy, PKC/ERK/CREB pathways, PSD-95, AMPAR/NMDAR). Search terms followed MeSH headings: bisphenol A, neurotoxicity syndromes, central nervous system, and prefrontal cortex. Boolean operators AND and OR were used to construct the combinations listed in Table 1.

Regarding the eligibility criteria established, inclusion: original experimental study (in vitro/in vivo) with BPA alone or BPA ± one isolable modulator; neural models (neural cells and/or cortical/hippocampal tissues); quantitative cellular/synaptic outcomes; PFC/hippocampus or neural cultures with plasticity endpoints; publication period 2007–2025; full text available; languages English, Portuguese, or Spanish.

Exclusion: reviews, editorials, and letters; epidemiological/observational studies without cellular/synaptic analysis; multiple co-exposures when the BPA effect could not be isolated; non-neural models; behavior-only studies without cellular correlation; no full text; outside the period/language limits; duplicates; misaligned with the study objective; and grey literature.

**Figure 1:**
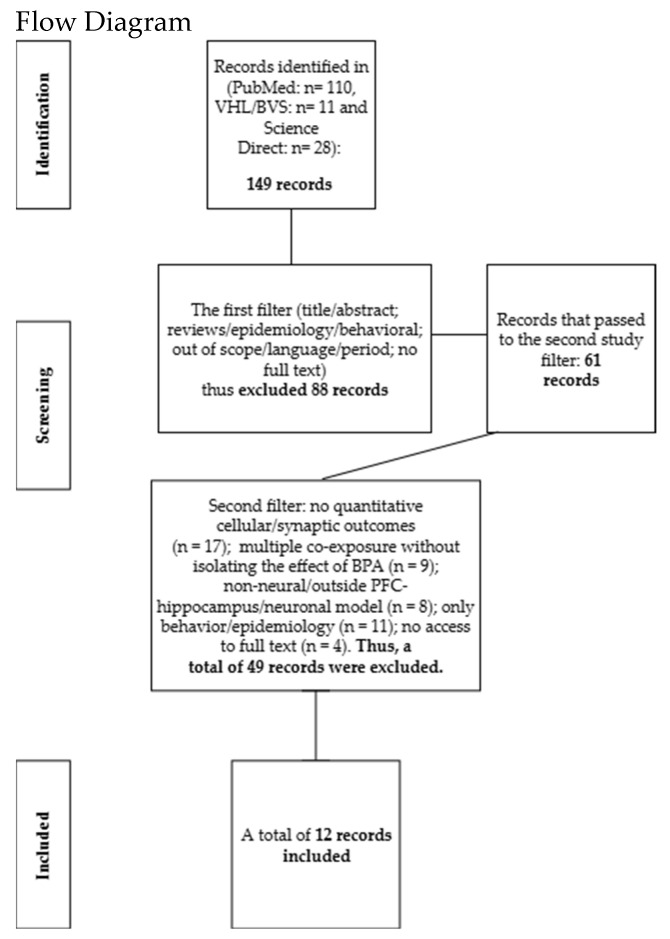
149 records identified; first screening excluded 88 (61 advanced). Second screening excluded 49 for methodological and scope reasons. Final sample: 12 studies included.

Screening was conducted on the Rayyan (QCRI) platform with three reviewers. Each reviewer was blinded to the others’ decisions. In cases of impasse or a tie, the final adjudication was referred to a third reviewer. In total, 149 articles were identified from PubMed (*n* = 110), the Virtual Health Library (VHL/BVS) (*n* = 11), and ScienceDirect (*n* = 28). In the first filtering stage, which involved the initial screening of titles and abstracts, 88 records were excluded for the following reasons: reviews; studies exclusively epidemiological or behavioral in nature; out of scope; language or time-frame restrictions; and lack of full text. After this stage, 61 articles remained. In the second filtering stage, which consisted of full-text assessment of these 61 articles, 49 were excluded for the following reasons: absence of quantitative cellular or synaptic outcomes (*n* = 17); multiple co-exposures without isolating the effect of BPA (*n* = 9); non-neural model or outside the PFC–hippocampus/neuronal scope (*n* = 8); behavioral or epidemiological focus only (*n* = 11); and no access to full text (*n* = 4). Consequently, 12 studies were included, as shown in the flow diagram below (Figure 1). The included studies are presented in Section 3.

## 3. Results

With regard to the results presented in this mini-review, a summary table was prepared compiling data from in vitro studies and research involving neuronal structures. As shown in Table 2, the selected articles were organized according to the following criteria: author/year, methodology, BPA dosage, neurotoxicity, and conclusion.

### 3.1. Bisphenol A-Induced Neurotoxicity: Evidence from Cell Cultures

This section addresses bisphenol A (BPA)-induced neurotoxicity in neural cell cultures. In this context, the experiment conducted by Wang et al. [14] primarily aimed to investigate the effects of chronic BPA exposure on glutamatergic neurons derived from human stem cells. To this end, the authors cultured these neurons and applied an increasing sequence of BPA concentrations (0.1, 0.5, 1, 5, and 10 μM). Additionally, dimethyl sulfoxide (DMSO, 0.1%) was used as the control solution. The exposure period ranged from 3 to 14 days, with the first three days designated for the assessment of short-term effects and 14 days for the analysis of long-term effects, representing continuous exposure to BPA.

From a morphological perspective, neurite outgrowth progressively decreased as BPA concentrations increased. In parallel, there was a significant reduction in cell viability at concentrations between 0.5 and 10 μM. Doses equal to or greater than 1 μM induced dendritic degeneration as well as cell body enlargement. Functionally, there was a marked upregulation of synaptophysin (SYN) and PSD-95, changes commonly associated with neuropsychiatric disorders. In addition, increased expression of AMPA receptors and the GRIP1 protein was observed, accompanied by a reduction in GluA2, which led to elevated intracellular calcium levels and the activation of cleaved caspase-3, a typical marker of apoptosis. Finally, the tested BPA concentrations promoted increased production of reactive oxygen species (ROS) and reduced antioxidant markers, contributing to heightened oxidative stress and cellular impairment.

Consistent with these findings, the experiments conducted by Lee et al. [15] sought to explore the mechanisms underlying BPA neurotoxicity, with particular emphasis on cell viability and neuronal differentiation. For this purpose, N2a cells, which possess a high differentiation potential, were employed. BPA concentrations of 0–9 μM, 10 μM, and 100 μM were tested. All concentrations were evaluated for 24 h, and the 100 μM dose was additionally assessed at early time points (5 to 60 min) and extended up to 14 days. At concentrations between 0 and 9 μM, no significant alterations or evidence of BPA-induced toxicity were observed. However, at doses ≥ 10 μM, a decline in cell viability became evident. At 100 μM, the most pronounced effects were observed, including marked viability loss, increased cellular debris, decreased culture density, impaired and fragmented neurites, activation of the apoptotic cascade, accumulation of autophagic vesicles, substantial ROS elevation, and reduced antioxidant activity. The authors concluded that BPA induces progressive, dose-dependent neuronal degeneration, confirming its neurotoxic potential.

Similarly, Cho et al. [16] evaluated the neurotoxic effects of BPA in neural tissues using primary cortical neurons. These cells were exposed to BPA at 50, 100, and 200 μM after seven days of culture for a period of five days. Concentrations of 50 and 100 μM did not induce evident cytotoxicity, whereas 200 μM resulted in neuronal death, marked impairment of neurite outgrowth, reduced cell viability, loss of mitochondrial membrane potential, and increased intracellular ROS levels. The authors concluded that 200 μM BPA exerts clear and severe neurotoxic effects.

**Figure 2:**
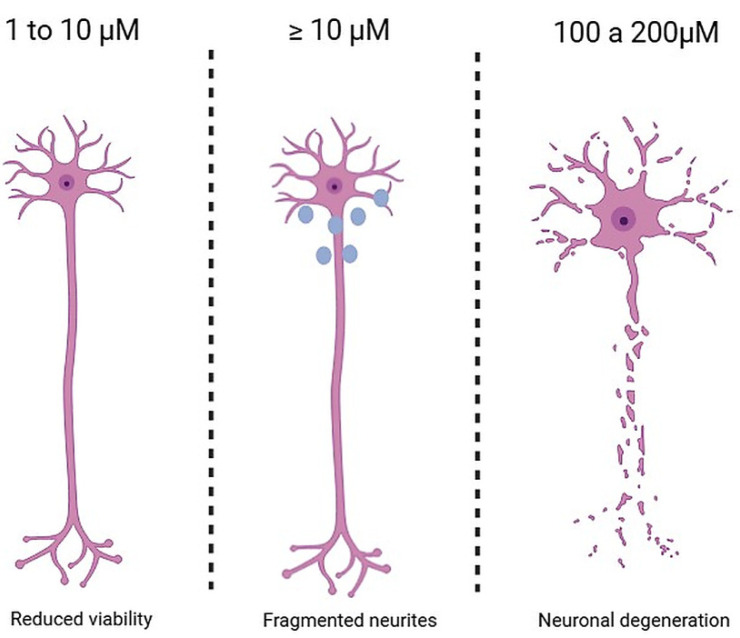
Effects of BPA dose on neuronal tissue. Legend: The figure illustrates, from left to right, the progressive neurotoxic effects of BPA. At 1–10 μM, neural cells exhibit reduced viability or minimal toxic alterations; at concentrations ≥ 10 μM, neurite fragmentation and impaired outgrowth appear; at 100–200 μM, there is pronounced neuronal degeneration and significant neurotoxicity.

Along the same line, Yin et al. [17] investigated neural plasticity and cytoskeletal integrity in Neuro-2a cells exposed to BPA. Cultures were divided into three groups: a negative control (culture medium), a DMSO control (0.001%), and treatment groups exposed to BPA (50, 100, 150, and 200 μM) for 24 h. Samples treated with 50 and 100 μM showed no significant changes in viability or morphofunctional parameters. However, at 150 μM, there was a notable reduction in cell viability, along with loss of membrane integrity, mitochondrial and nuclear alterations, and decreased levels of key markers such as SYP, drebrin (Dbn), MAP2, and Tau. At 200 μM, these effects were even more pronounced, culminating in extensive neuronal death, see Figure 2.

Moving toward a broader perspective on neural structures and functions, Zhang et al. [13] investigated the effects of low BPA doses on neural development in postnatal rodents. Animals were exposed to BPA via intraperitoneal injections at low (0.5 μg/kg), moderate (50 μg/kg), and high (5000 μg/kg) doses, in addition to a non-exposed control group. To evaluate complex neural structures and functions, such as those of the hippocampus, the authors performed Y-maze testing to assess memory and Golgi-Cox staining to analyze dendritic morphology. The results revealed that low and moderate doses caused impairments in learning and memory and reduced dendritic complexity, while high doses produced severe memory deficits and almost complete loss of dendritic growth.

Supporting these findings, Wu et al. [12] investigated whether BPA exposure increases oxidative stress and consequently impairs cognitive function. BPA was administered in drinking water at 0.1 μg/mL and 0.2 μg/mL for eight weeks. Animals underwent behavioral tests, including the Morris water maze, object recognition, and the shuttle box, to assess memory. The results showed that 0.1 μg/mL exposure caused mild spatial memory impairments, while 0.2 μg/mL led to pronounced deficits in spatial memory, recognition, and avoidance learning. These findings reinforce those of Zhang et al., confirming that even very low BPA doses impair learning and memory.

Finally, Kim et al. [11] examined the neurotoxic effects of BPA on the rodent hippocampus. Animals were exposed to BPA for two weeks at 1, 5, and 20 mg/kg/day. Analyses included proliferation and survival of new cells in the dentate gyrus, cerebral and hippocampal perfusion, and spatial learning and memory testing. The results demonstrated that 20 mg/kg/day BPA caused substantial cellular loss in the hippocampus and severe impairments in memory and learning.

Advancing to studies involving low BPA concentrations aligned with the European Food Safety Authority (EFSA) tolerable daily intake (TDI, ~0.2 ng/kg/day), the experiment by Kiso-Farnè et al. [18] aimed to determine whether low BPA doses disrupt corticogenesis and neuronal differentiation in human neural stem cells. Human neural stem cell models were exposed to 0.1, 1, 10, and 100 nM for 2, 4, 7, and 12 days in vitro, followed by immunocytochemistry and quantitative cell analysis. The findings indicated altered cellular differentiation and proliferation, especially at 100 nM, where there was a significant reduction in SOX2 after 4–7 days and notable morphological changes.

Similarly, Pang et al. [20] compared the toxicity of BPA and its analogues (BPS and BPB) in murine hippocampal neurons, focusing on oxidative stress, apoptosis, and cell proliferation. Concentrations ranged from 1 nM to 100 μM, with oxidative stress assessed after 6 h, apoptosis after 24–48 h, and proliferation after 7 days. The study showed that even low doses increased oxidative stress; BPS was the least toxic, while BPA and BPB significantly reduced proliferation and triggered cell death at higher doses.

Corroborating these findings, Liang et al. [21] examined BPA and six derivatives (BPS, BPE, BPF, BPB, BPAF, and BPZ) in human neuronal cells, investigating viability, differentiation, and neurite morphology. Cell viability was tested at 0.001–300 μM for 24 h, while neurite length and differentiation were assessed at 1, 10, and 100 nM for eight days. Toxicity was notable only at high concentrations (>25 μM); however, even low doses (1–100 nM) significantly reduced neurite length, suggesting functional impairment without overt cell death. Among the derivatives, BPAF and BPB were the most toxic, whereas BPS was the least harmful.

Finally, Flores et al. [19] investigated the impact of BPA on the cholinergic system, including neuronal death, neural plasticity, and signaling pathways. Both in vivo and in vitro experiments were conducted. In male rodents, a single dose of 40 μg/kg BPA was administered and evaluated after 48 h, revealing selective neuronal loss and necrosis in cholinergic nuclei. In vitro, basal forebrain cholinergic neurons were exposed to 0.001–1 μM BPA for 1–14 days, demonstrating dose-dependent cell death, marked degeneration, loss of synaptic proteins, and dysfunction of cholinergic and glutamatergic pathways.

### 3.2. Teratogenic Potential of Bisphenol A in the Central Nervous System and Its Interference with Synaptic Plasticity in the Prefrontal Cortex and Hippocampus

Experimental studies in animal models have demonstrated that prenatal exposure to estrogenic endocrine disruptors, such as bisphenol A (BPA), is associated with significant teratogenic effects. This has raised increasing concern within the fields of neuroscience and environmental neurotoxicology regarding the implications of BPA for neural development [3].

Xing et al. [22] reported that BPA, either alone or in combination with genistein, induces neural tube malformations in rat embryos, particularly affecting the prosencephalon. These effects were dose-dependent and revealed a negative synergistic interaction between the compounds, amplifying damage to central nervous system (CNS) development. Converging lines of evidence indicate that BPA, a widely used endocrine disruptor, possesses teratogenic potential in the CNS, particularly by impairing synaptic plasticity in the prefrontal cortex and hippocampus [23,24,25]. Exposure to BPA during critical periods of neurogenesis may disrupt neuronal migration and maturation, leading to persistent morphofunctional alterations and an increased risk of neuropsychiatric disorders [26].

BPA exposure during vulnerable developmental windows, such as gestation and early childhood, has been linked to morphological and functional changes in the CNS. Studies have shown that BPA reduces neuronal populations while increasing glial cell numbers in the cerebral cortex and impairs the self-renewal and differentiation of neural progenitors, leading to long-lasting behavioral and cognitive deficits [23]. In the hippocampus, BPA interferes with synapse formation and maintenance, reduces dendritic spine density, and impairs synaptic transmission, resulting in memory and learning impairments [6,24,27].

GABAergic neurotransmission, essential for emotional and psychophysiological regulation, is also disrupted by BPA exposure. BPA has been shown to increase the expression of cytochrome P450 and tryptophan hydroxylase genes in the prefrontal cortex while reducing 5α-reductase expression in females, indicating a sexually dimorphic response in adult rats [25]. Moreover, BPA decreases mRNA levels of CaMKII subunits, a protein critical for memory consolidation and synaptic plasticity [28].

From a pathophysiological perspective, BPA has been implicated in the suppression of potassium-chloride cotransporter 2 (KCC2) gene expression, essential for the developmental switch in GABAergic activity from excitatory to inhibitory. This suppression may compromise synaptic organization and cortical architecture [29]. BPA also disrupts both pre- and postsynaptic mechanisms, decreasing glutamate release, reducing dendritic spine density, and downregulating NMDA and AMPA receptors, which are vital for synaptic plasticity and long-term potentiation (LTP). In the prefrontal cortex, BPA blocks the synaptogenic response normally induced by sex hormones such as testosterone and estradiol, preventing the physiological synaptic increase these steroids promote [6,25,30]. Reported that BPA blocks estradiol-dependent memory consolidation in ovariectomized female rats, leading to object recognition deficits.

In nonhuman primates, even low BPA levels, comparable to typical human exposure, have been shown to reduce excitatory synapses and impair working memory, with partial recovery after compound withdrawal [25]. Brain estrogens, derived from androgens, play a fundamental role in neural development [31], yet BPA disrupts the development of midbrain dopaminergic neurons and hippocampal synapses in rodents [32,33]. In primates, gestational BPA exposure has resulted in abnormalities in the fetal ventral midbrain and hippocampus. In juveniles, however, no morphological or cognitive alterations were detected, suggesting that BPA’s effects are developmental stage-dependent [25]. Furthermore, BPA acts as a modulator of estrogen receptors, exhibiting both agonistic and antagonistic activity, and displays antiandrogenic properties, further disrupting synaptic plasticity [5].

At the molecular level, BPA alters intracellular signaling pathways, including the NMDAR/PSD-95–PTEN/AKT axis, thereby affecting the expression of synaptic proteins and transcription factors linked to autism spectrum disorders and neuroplasticity. These molecular alterations are associated with anxiety- and depression-like behaviors, cognitive impairments, and a potentially increased risk of autism spectrum disorders, particularly following perinatal exposure [34,35].

Given this evidence, it is crucial to prioritize research employing long-term, low-dose exposure models that more accurately reflect human environmental contact with BPA and its analogues (e.g., BPF, BPAF, and BPS). Such approaches would better simulate continuous, lifelong exposure scenarios. Additionally, there is a clear need to use advanced human-relevant models, including brain organoids, induced pluripotent stem cells (iPSCs), and multi-omics technologies (transcriptomics, epigenomics, metabolomics), to identify early neurodevelopmental alterations and behavioral outcomes. Equally important is the expansion of epidemiological studies to improve biomonitoring and cumulative risk assessment, especially in relation to real-world exposure levels, including concentrations below the recently revised tolerable daily intake (TDI) set by EFSA (0.2 ng/kg/day). Finally, future efforts should focus on exploring neuroprotective strategies and assessing the comparative safety of BPA substitutes, thereby supporting stronger regulatory frameworks and more effective public health interventions.

## 4. Conclusions

Therefore, evidence from studies using both human neuronal cell cultures and animal models consistently indicates the neurotoxic potential of bisphenol A (BPA). This compound exerts deleterious effects by disrupting cellular structure and function across a wide range of exposure levels. Notably, human embryonic neuronal cells and animal neural systems exposed to concentrations between 1 and 10 μM exhibit morphological alterations, including impaired neuritic outgrowth, neurite fragmentation, and dendritic retraction. At doses ≥ 100 μM, BPA becomes markedly cytotoxic, triggering cellular apoptosis via apoptosis-inducing factor (AIF) in a caspase-3-independent manner and blocking autophagic flux through the HO-1/AMPK pathway. In addition, oxidative stress is exacerbated, accompanied by dysregulation of calcium homeostasis, collectively leading to significant neuronal damage and weakening of synaptic integrity and plasticity.

These converging findings clearly demonstrate that BPA-induced cellular injury leads to nervous system dysfunction, including hippocampal impairment and cognitive deficits. Of particular note, exposures between 1 and 50 μM for 14 days or longer reduce hippocampal neurogenesis and severely impair spatial memory. At ≥100 μM, the neurotoxic effects are more pronounced, resulting in extensive neuronal death.

In summary, this mini-review underscores that BPA is a concentration-dependent neurotoxicant: lower doses produce subtle but measurable neural alterations, whereas higher doses lead to profound structural and functional damage. Importantly, BPA can compromise neural architecture, intracellular signaling, and memory processes. Future research should therefore prioritize standardized, long-term exposure models and robust behavioral assessments in both experimental systems and humans to strengthen causal inference and inform regulatory safety thresholds.

## Figures and Tables

**Table 1:** Search strategy.

Descriptors and Combinations
**1#**—Diphenylolpropane OR 2,2-bis(4-hydroxyphenyl)propane OR 4,4′-dihydroxy-2,2-diphenylpropane OR bisphenol A, sodium salt OR bisphenol A, disodium salt OR disodium bisphenol A
**2#**—Neurotoxicity Syndrome OR Syndrome, Neurotoxicity OR Syndromes, Neurotoxicity OR Poisoning, Nervous System OR Nervous System Poisonings OR Poisonings, Nervous System OR Nervous System Poisoning OR Neurotoxic Disorders OR Neurotoxic Disorder OR Neurotoxin Diseases ORNeurotoxin Disease OR Neurotoxin Disorders OR Neurotoxin Disorder OR Toxic Encephalitis OR Encephalitides, Toxic OR Encephalitis, Toxic Toxic Encephalitides OR Encephalopathy, Toxic OR Encephalopathies, Toxic OR Toxic Encephalopathies OR Toxic Encephalopathy.
**3#**—Central Nervous Systems OR Nervous System, Central OR Nervous Systems, Central OR Systems, Central Nervous OR Cerebrospinal Axis OR Axi, Cerebrospinal OR Axis, Cerebrospinal OR Cerebrospinal Axi.
**4#**—Cortex, Prefrontal OR Frontal Sulcus OR Sulcus, Frontal OR Superior Frontal Gyrus OR Frontal Gyrus, Superior OR Gyrus, Superior Frontal OR Marginal Gyrus OR Gyrus, Marginal OR Gyrus Frontalis Superior OR Frontalis Superior, Gyrus OR Superior, Gyrus Frontalis OR Superior Frontal Convolution OR Convolutions, Superior Frontal OR Convolution, Superior Frontal OR Frontal Convolution, Superior OR Superior Frontal Convolutions OR Medial Frontal Gyrus OR Frontal Gyrus, Medial OR Gyrus, Medial Frontal OR Inferior Frontal Gyrus OR Frontal Gyrus, Inferior OR Gyrus, Inferior Frontal OR Gyrus Frontalis Inferior OR Inferior, Gyrus Frontalis OR Straight Gyrus OR Gyrus, Straight Brodmann’s Area 11 OR Area 11, Brodmann’s OR Brodmanns Area 11 OR Gyrus Rectus OR Rectus Gyrus OR Gyrus, Rectus OR Rectal Gyrus OR Gyrus, Rectal OR Brodmann Area 11 OR Area 11, Brodmann OR Orbitofrontal Cortex OR Cortex, Orbitofrontal OR Orbitofrontal Cortices OR Orbitofrontal Gyrus OR Gyrus, Orbitofrontal OR Orbitofrontal Gyri OR Orbital Cortex OR Cortex, Orbital OR Orbital Cortices OR Orbital Area OR Area, Orbital OR Orbital Areas OR Gyrus Orbitalis OR Orbital Gyri OR Gyrus, Orbital OR Orbital Gyrus OR Orbitofrontal Region OR Orbitofrontal Regions OR Region, Orbitofrontal OR Subcallosal Area OR Area, Subcallosal OR Subcallosal Areas OR Ventromedial Prefrontal Cortex OR Cortex, Ventromedial Prefrontal OR Cortices, Ventromedial Prefrontal OR Prefrontal Cortex, Ventromedial OR Ventromedial Prefrontal Cortices OR Ventral Medial Prefrontal Cortex OR Olfactory Sulci OR Olfactory Sulcus OR Lateral Orbitofrontal Cortex OR Cortex, Lateral Orbitofrontal OR Lateral Orbitofrontal Cortices OR Orbitofrontal Cortex, Lateral OR Orbitofrontal Cortices, Lateral OR Anterior Prefrontal Cortex OR Anterior Prefrontal Cortices OR Cortex, Anterior Prefrontal OR Prefrontal Cortex, Anterior OR Prefrontal Cortices, Anterior OR Brodmann Area 10 OR Area 10, Brodmann OR Brodmann’s Area 10 OR Area 10, Brodmann’s OR Brodmanns Area 10 OR Brodmann Area 12 OR Area 12, Brodmann OR Brodmann’s Area 12 OR Area 12, Brodmann’s OR Brodmanns Area 12 OR Brodmann Area 47 OR Area 47, Brodmann OR Brodmann’s Area 47 OR Area 47, Brodmann’s OR Brodmanns Area 47 OR Pars Orbitalis OR Orbitalis, Pars.

Source: author (2025).

**Table 2:** Compendium.

Author/Year	BPA Dosages	Neurotoxicity	Results
Kim et al. [11]	1, 5, and 20 mg/kg/day	Neurogenesis, BDNF, ROS, cognition.	20 mg/kg ↓ new hippocampal cells & spatial memory; low dose ↑ cell survival without cognitive gain; no neuronal loss or astrocyte activation
Wu et al. [12]	0.1–0.2 μg/mL BPA ± 0.6 mg/mL ALA.	Cognition, oxidative stress, synaptic proteins, PKC/ERK/CREB.	BPA impaired multiple memory types, ↓ neurotransmitters & synaptic proteins; induced oxidative stress. ALA reversed these effects.
Zhang et al. [13]	0.5, 50, and 5000 μg/kg/day	Learning/memory, dendritic complexity, neurotransmitters.	Low & high doses impaired spatial learning, reduced dendritic complexity/spine density; sex-specific neurotransmitter shifts (↑ Glu/ACh, ↓ GABA/5-HT in males).
Wang et al. [14]	0.1–10 μM	Reduced neurite outgrowth, synaptic disruption, apoptosis.	BPA reduced neurite length, disrupted dendritic spines, increased synaptic proteins, and triggered oxidative/nitrosative stress with calcium imbalance.
Lee et al. [15]	10–100 μM	Autophagy and apoptosis.	BPA ≥ 100 μM ↓ viability & axon growth; AIF-driven apoptosis and blocked autophagic flux; HO-1/AMPK involved.
Cho et al. [16]	1–100 μM	Early neuronal differentiation.	Standard assays only detected toxicity ≥ 200 μM; DCX/MAP2 ICC revealed impaired maturation at 100 μM.
Yin et al. [17]	1–100 μM	Synaptic and cytoskeletal damage.	Dose-dependent ↑ cell death, synapse loss, cytoskeleton injury (↓ MAP2/Tau/Dbn, ↑ SYP); mitochondrial/nuclear alterations.
Kiso-Farnè et al. [18]	0.1; 1; 10; 100 nM	Corticogenesis disruption.	100 nM ↓ stem cells & ↑ immature neurons; morphological shift to multipolar cells.
Flores et al. [19]	40 µg/kg (rats); 0.001–1 µM (cells)	Basal forebrain cholinergic degeneration.	BPA caused neuronal loss, PSD95/SYP ↓, glutamate ↑, WNT/β-catenin disruption, HDAC2-driven cell death.
Pang et al. [20]	0.1–200 µM	Oxidative stress & cell death.	BPA ↑ ROS/apoptosis and ↓ proliferation; non-monotonic dose–response.
Liang et al. [21]	Nanomolar to micromolar (0.024–50 nM, up to µM)	Reduced neurite length and impaired human neuronal development.	BPA and its analogues shortened neurite outgrowth even at very low concentrations; BPAF most toxic, BPS least.
Xing et al. [22]	BPA: 0–32 μg/mL; GEN: 0–10 μg/mL	Severe CNS malformations	BPA alone minimally teratogenic; co-exposure with GEN significantly enhanced embryotoxicity and neural malformations.

Source: Author (2025). Legend: the abbreviations used in this Table 2 correspond to the following terms: ALA = alpha-lipoic acid; AMPAR = α-amino-3-hydroxy-5-methyl-4-isoxazolepropionic acid receptor; AIF = apoptosis-inducing factor; ACh = acetylcholine; BDNF = brain-derived neurotrophic factor; BPA = bisphenol A; BPAF = bisphenol AF; BPF = bisphenol F; BPS = bisphenol S; CREB = cAMP response element-binding protein; CNS = central nervous system; DCX = doublecortin; Dbn = drebrin; ERK = extracellular signal-regulated kinase; GABA = gamma-aminobutyric acid; Glu = glutamate; GEN = genistein; HDAC2 = histone deacetylase 2; HO-1 = heme oxygenase-1; ICC = immunocytochemistry; MAP2 = microtubule-associated protein 2; PSD95/PSD-95 = postsynaptic density protein 95; PKC = protein kinase C; ROS = reactive oxygen species; RNS = reactive nitrogen species; SYP = synaptophysin; SOX2 = SRY-box transcription factor 2; Tau = microtubule-associated protein tau; WNT/β-catenin = Wingless/Integrated signaling pathway; ↓ Reduction in the index.

## Data Availability

This manuscript is a mini-review; no new data were generated or analyzed. All information supporting the reported results derives from previously published studies, cited in the References and accessible through public repositories/databases (e.g., PubMed, Virtual Health Library [BVS], and ScienceDirect). No additional datasets are available.
